# Treatment of displaced supracondylar fracture of the humerus in children by open pining from lateral approach: an investigation of clinical and radiographical results

**DOI:** 10.12669/pjms.314.7696

**Published:** 2015

**Authors:** Nasser Sarrafan, Seyed Abdolhossein Mehdi Nasab, Tahmineh Ghalami

**Affiliations:** 1 Nasser Sarrafan, Associated Professor of Orthopaedic Surgery, Department of Orthopaedics, Emam Khomeini Hospital, Ahvaz Jondishapur University of Medical Sciences, Ahvaz Iran; 2 Seyed Abdolhossein Mehdi Nasab, Professor of Orthopaedic Surgery, Department of Orthopaedics, Emam Khomeini Hospital, Ahvaz Jondishapur University of Medical Sciences, Ahvaz Iran; 3 Tahmineh Ghalami, Orthopaedic surgeon, Department of Orthopaedics, Emam Khomeini Hospital, Ahvaz Jondishapur University of Medical Sciences, Ahvaz Iran

**Keywords:** Humerus supracondylar fracture, Open reduction, Lateral pinning

## Abstract

**Background and Objective:**

Supracondylar fracture of the humerus is the most common elbow fracture in children. This fracture needs immediate diagnosis and treatment, otherwise, it may lead to significant neurovascular and functional problems. The aim of this study was to assess the short term outcome of displaced supracondylar fracture of the humerus in children by open reduction and pining from lateral approach.

**Methods:**

During a period of 15 months from June 2012 to September 2013, 48 patients (25 boys and 23 girls) less than 10 years old were enrolled in the study. Inclusion criteria were extension type supracondylar fractures of humerus, Gaartland type III that closed reduction was unsuccessful and failed as the initial treatment. The clinical and radiographic results of the treatment using open reduction and internal fixation by lateral pinning were evaluated. Outcomes were assessed according to the Flynn’s criteria.

**Results:**

The average age of the patients was 6.3 years. The most prevalent range of age was found about 6-9 years old. All patients had extension type fracture (Gartland type III). Overall, 47 (98%) patients had closed fracture and only one (2%) had open fracture. Eighteen patients (37.5%) and 30 patients (62.5%) had involvement of the dominant and non-dominant extremity respectively. No vascular injury and infection was seen in patients. One patient (2%) was identified with the radial nerve injury which, recovered after three months. In the three and six month follow-up, one patient (2%) was found with the median nerve injury. Since 15 patients were lost to follow-up, the analysis of the clinical and radiographical results at the end of the 6^th^ month were done for 33 patients. According to the Flynn’s criteria, the cosmetic results in 30 out of 33 patients that completed their follow-up (90.09%) were excellent, in 2 patients (6.1%) were good and one case (3%) was fair (P=0.051). Also, the functional results in 31 patients (93.9%) were excellent and in 2 patients (6.1%) were good. Overall, all cases were graded satisfactory (P=0.047).

**Conclusions:**

Treatment of the supracondylar humeral fracture in children by open reduction and internal fixation through lateral pinning is a safe approach with predictable good clinical and radiographical results.

## INTRODUCTION

Humerus supracondylar fracture is the most common elbow fracture in children that it includes about 60% of the elbow fractures and 13%-15% of all pediatric fractures.^1^,^2^ This fracture is more common in 5-7 years old children. Also, boys are more affected by this fracture in comparison with girls.^2^,^3^ The prevalence of this fracture decreases after 12 years age.^1^ This fracture often occurs in left side or the non-dominant side.^4^,^5^ Supracondylar fracture is generally classified as the flexion and extension types. About 97-99% of cases are of the extension type.^2^,^6^ The most common mechanism is falling on outstretched hand while, elbow is in extension. The extension type fracture is classified according to the standard Gartland classification in which, Type I is non-displaced, Type II is displaced but posterior context intact and Type III is completely displaced fracture. This type of fracture is unstable and may leads to severe complications and require operative treatment.^7^

In extension type fractures, the radial and median nerve and brachial artery are more prone to injury. This is while, in the flexion type fractures, the ulnar nerve injury is more probable.^8^ Forearm fracture along with supracondylar fracture increases the risk of compartment syndrome. The prevalence of vascular injury in fracture around the elbow is estimated about 12-20%.^9^,^10^ The incidence of traumatic and iatrogenic nerve injuries with this fracture have been recorded as 12%–20% and 2%–6%, respectively.^11^ The open method is recommended in cases of open fracture, failed or impossible closed reduction and also when the vascular injury is probable and the supracondylar fracture is in company with the forearm fracture. There are many operative techniques for ORIF in humerus supracendylar fractures such as percutaneaus fixation, mediolateral pin fixation and lateral pin fixation and posterior approach.

One technique of ORIF is from lateral approach, because less soft tissue dissection is needed and damage of ulnar nerve will be prevented with this technique. For cases who need ORIF, lateral approach is a less invasive with minimum soft tissue dissection in comparative with posterior approach. This later is associated with triceps muscle dissection or splitting, and more post operative adhesion.^12^,^13^

The present work in context of a prospective study aims at investigating the clinical and radiographical results of the treatment of the supracondylar fracture by the lateral pining method (open approach).

This study was aimed to assess the short term outcome of displaced humerus supracondylar fractures by open reduction and internal fixation through lateral approach to evaluate both clinical and radiographic results of humerus supracondylar fractures.

## METHODS

In this prospective study, 48 patients less than 10 years of age were enrolled. First, all patients diagnosed as the supracondylar fracture in Imam Khomeini and Razi hospitals of Ahvaz, during June 2012 and September 2013, were considered. Inclusion criteria was all the patients with type III unstable supracondylar humerus fracture who closed reduction was failed and need open reduction & internal fixation.

The patients were characterized by means of the Gartland’s classification method. Patients with fracture type III and treated by open reduction method were taken into account in this study. Among these patients, no case had flexion type fracture.

All patients had extension type fracture (type III). 30 patients (62.5%) was operated in 24 hours after injury while, 18 patients (37.5%) was operated after 24 hours. Overall, 47 (98%) patients had closed fracture and only one (2%) had open fracture.

In general, for the patients with the supracondylar fracture, closed reduction method is preferred. However, for the patients used in this study, closed reduction was not either possible or was tried first but failed. Initial closed reduction was performed in operation room under C-ARM control. The conversion of closed to open technique was due to inability to gain anatomic reduction in these unstable fractures. We did not use medio-lateral fixation by posterior approach, because it is a major surgery with dissection or splitting of triceps muscle.

For all the patients ORIF was performed under general anesthesia, tourniquet inflation, and lateral approach with protection of radial nerve, internal fixation by two (29 cases) or three (19 cases) lateral pins, which were placed in parallel fasion. Patients were followed up for 6 months. However, 15 out of 48 patients quitted the program. These patients were excluded from the final analysis (Fig. 1A and 1B).

**Fig.1A:**
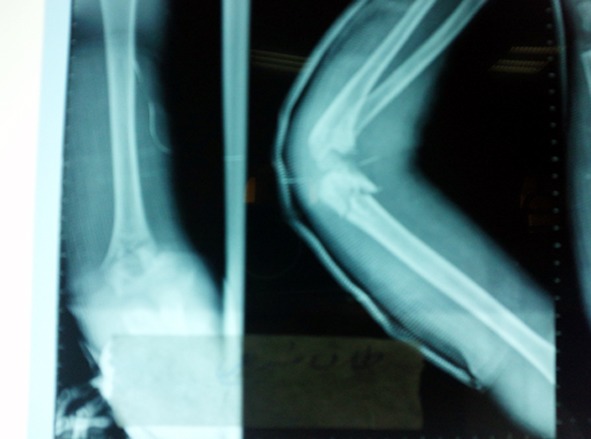
Displaced humerus supracondylar fracture in a 5 years old patient.

**Fig.1B:**
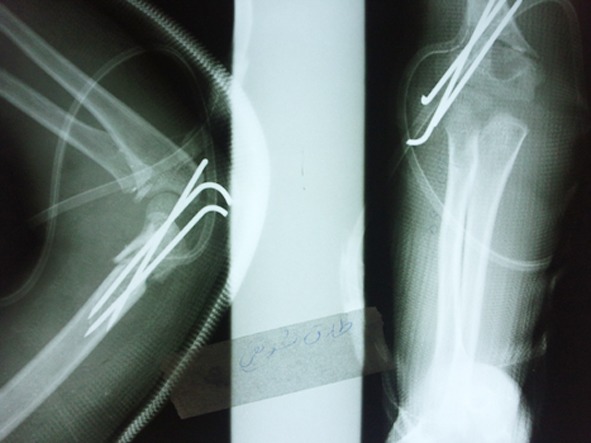
Post op. elbow radiography.

The control radiographies were performed for the first, second, third and fourth weeks and then, for the third and sixth months after operation. In cases where the patient was treated by open reduction and internal fixation, the surgical approach was through the lateral incision on the lateral condyle with a mild tilt toward the anterior. The fracture was fixed by using a few number of pins (depending on the fracture type) from lateral. The pins were bended and kept outside the skin and the extremities were maintained in a splint with 90° flexion. All patients were hospitalized for 8 hours. Also, the patients were given the oral prophylactic antibiotic for 3-5 days and the sutures were removed after 14 days. For all patients, the splints and pins were removed by the end of forth week after operation at which, the union was completed. Criteria for radiographic union was based on disappearance of fracture site and to see callus formation in at least 3 cortex

**Fig.2:**
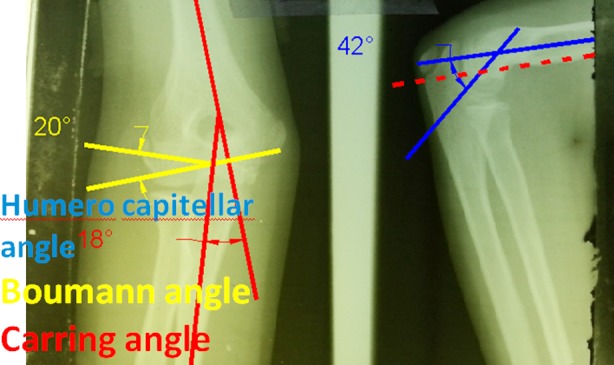
AP and LAT views of elbow cubitus valgus after union of humerus supracondylar fracture.

Afterwards, the patients and their parents were educated to initiate the elbow range of motion exercises at home. The patients who had limited range of motion of the elbow at the end of the second month, were referred to the physiotherapist. The clinical evaluations were performed for all patients at the end of the sixth month. The flexion, extension, supination and pronation were investigated and the limitation of each was recorded in degree. The clinical evaluation of angular deformity (varus or valgus) was performed and the carrying angle in both sides was measured by goniometer. The radiographic assessment was carried out after the sixth month by investigating the humeroulnar angle and Baumann angle in AP view and, humerocapitellar angle in LAT view (Fig.2). The results were evaluated by the Flynn’s criteria as presented in Table-I. These criteria are very helpful for investigating the results of the supracondylar fractures. By these criteria, the patients are evaluated by means of the functional and cosmetic factors. On this basis, the functional evaluations were done using the degree of limitation of range of motion and the cosmetic evaluations were done using the measurement of the carrying angle in both sides. This study was approved by ethics committee at our university, and an informed consent was taken as a routine base at the time of admission.

**Table-I:** Frequency of the patients with respect to the involvement side.

Sexuality	No. of left extremity	No. of right extremity	No. of dominant extremity	No. of non-dominant extremity
Boys	13	12	11	14
Girls	16	7	7	16

This study was approved by the Ethics Committee of our university and, an informed consent was taken from the patients.

## RESULTS

The mean age of the patients was 6.3 years old (ranging from 1.5 to 11.5) among which, 25 patients (52.1%) were boy and 23 (48%) were girl. The average age of boys and girls was respectively 6.9 and 5.7 years old. The most prevalent range of age in this study, for both girls and boys, was found to be 6-9 years old which is in agreement with the previous investigations.

In 29 patients (60%), the left extremity was involved while, in the other 19 patients (40%) the right extremity was involved. According to the dominant and non-dominant extremity, 18 patients (37.5%) and 30 patients (62.5%) respectively, had involvement of the dominant and non-dominant extremity. The frequency of patients with respect to sex and side of the involvement is presented in Table-I.

The mechanism of injury in 19 patients (39.6%) was due to fall while playing, in 25 patients (52.1%) was fall from height and in 4 patients (8.33%) it was due to the motor vehicle accident. The mean operation time was estimated at about 43 minutes ranging from 15 to 60 minutes.

The internal fixation was done by using lateral pining in parallel fashion (2 or 3 pins). No post-operative complication such as vascular injury and infection was seen in the patients. During the first 4-week follow-up, one patient (2%) was identified with the radial nerve injury which, recovered after three months. In three and six months follow-up, one patient (2%) was found with the median nerve injury.

Since 15 patients were lost to follow-up, the analysis of the clinical and radiographical results at the end of the 6^th^ month were done for 33 patients. No revision surgery was performed because of inappropriate radiographic indexes.

For all 33 patients who completed their 6-month follow-up, the humeroulnar angle and Baumann angle in AP view and humerocapitellar angle in LAT view (Fig.2) were measured at the end of the 6^th^ month. Table-II shows the maximum, minimum and average values of the aforementioned angles. Fracture union occurred in all patients in 3- 5 weeks, (mean time of 4.2 weeks).

**Table-II:**
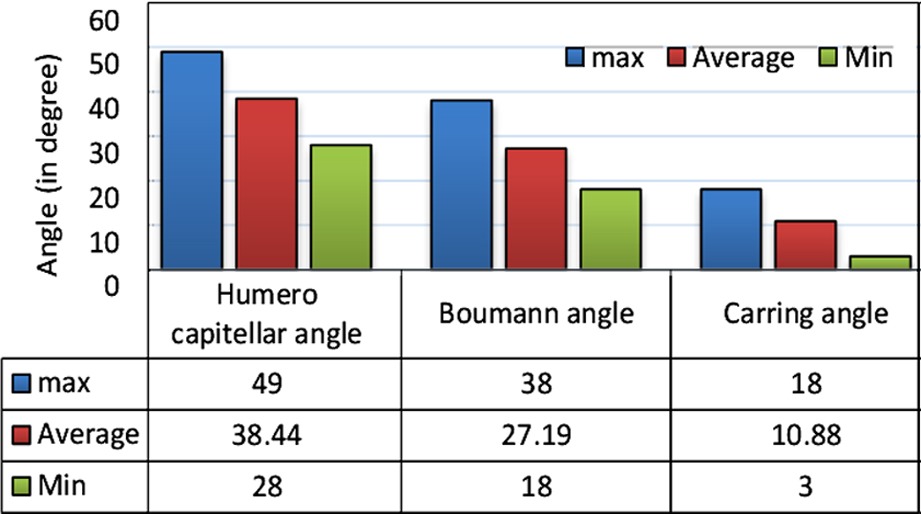
Measured angles (in degree) for the patients.

According to the Flynn’s criteria (Table-III), the cosmetic results in 30 out of 33 patients that completed their follow-up (90.09%) were excellent, in 2 patients (6.1%) were good and one case (3%) was fair (P=0.051). The functional results in 31 patients (93.9%) were excellent and in 2 patients (6.1%) were good (P=0.047). Overall, all cases were graded satisfactory as presented in Table-IV.

**Table-III:** Flynn’s criteria and overall rating.

Result	Grading	Cosmetic factor Carrying angle loss (degrees)	Functional factor Movement loss (degrees)	Overall rating
Satisfactory	Excellent	0 to 5	0 to 5	The lower of the two ratings and the elbow with a varus deformity is automatically graded as poor
	Good	6 to 10	6 to 10	
	Fair	11 to 15	11 to 15	
Unsatisfactory	Poor	>15	>15	

**Table-IV:** Final results according to the Flynn’s criteria.

Grading	Cosmetic factor: Carrying angle loss (Number of cases)	Functional factor: Movement loss (Number of cases)	Overall result	Percentage (%)
Excellent	30	31	30	90.9
Good	2	2	2	6.1
Fair	1	0	1	3
Poor	0	0	0	0

## DISCUSSION

Humerus supracondylar fracture is the most common elbow fracture in children mostly in range of 6-9 years, as found in this study. According to the Gartland Classification, type III of this fracture need to be treated by closed or open reduction and internal fixation. In treatment of the supracondylar fracture, the main target is to gain anatomic reduction functional with no serious complication. In case of fractures without displacement, almost all surgeons agree on the non-operative treatment. However for displaced fractures, several approaches might be chosen. In this context, a common approach for the open reduction is the pinning from lateral side of the elbow. According to the Flynn’s criteria, results showed that about 90.9% of the treatments had satisfactory results. However, it is worth noting that while, some surgeons initially adopt the open reduction approach as the treatment modality especially for type III, some others believe that this approach should be applied only if the closed reduction method was failed.^12^ The later opinion is acknowledged in this study^13^,. In an investigation by Weiland et al.^14^,^15^ it was concluded that short term results of open reduction was similar to the closed reduction method. However, they found that open reduction had less cubitus varus and valgus complications. In the present study, we found that open method has more anatomic reduction and satisfactory outcomes with a low rate of minor varus or valgus deformity.

Woratanarat et al.^16^ did a meta-analysis based on a plenty of articles on pinning methods. They concluded that lateral pinning outperforms the medial-lateral pining methods since it causes less ulnar nerve injury. Similar findings were also reported in Skaggs et al.^17^ and Gaston et al.^18^ The results of this work are also in agreement with the previous studies.

In a study by Zamzam et al.^19^ the patients treated by lateral pinning were prone to failure of fracture stability, complications and more re-operation rates.The previous investigations have shown that outcome of the lateral pinning approach is very satisfactory so that, about 67%-91.8% of treatments were found successful.^10^-^12^,^20^,^21^ The outcomes of this study also show that about 90.9% of treatments are excellent and according to the Flynn’s criteria all treatment results are satisfactory.

In spite of all the excellent results found here and in the previous studies on the use of lateral pinning method, there is no general agreement on the treatment approach for the supracondylar fracture. Indeed, excellent outcomes have also been seen in anterior, posterior and medial approaches. In other words, there is no dramatic statistical differences between the aforementioned approaches.^6^-^8^

In the previous studies, various methods have been proposed for pinning.^22^ However, cross pinning from medial and lateral has been found to be the most stable method in biomechanical standpoint.^5^,^7^ In our study, the lateral pinning by using 2 or 3 pins were successfully applied to the patients. Upon the results presented in the previous section, the demographic results, injury mechanism and the morphologic characteristics of the patients are in agreement with the previous studies. The limitation of the study was related to short term follow up. In addition \ most of our patients were referred late with massive swelling hencet closed reduction was unsuccessful for them. Another limitation was that a uniform and regular physiotherapy program was not available in the same center for all the patients.

## CONCLUSION

High rates of satisfactory results were found in the treatment of displaced supracondylar fractures of the humerus by lateral pinning method. According to the clinical and radiographical results, we conclude that the lateral pinning method is reliable and safe in terms of elbow function, neurovascular injury and infection issues.
